# Treatment of Pediatric *Helicobacter pylori* Infection

**DOI:** 10.3390/antibiotics11060757

**Published:** 2022-06-01

**Authors:** Hung-Hsiang Lai, Ming-Wei Lai

**Affiliations:** 1 Department of Pediatrics, New Taipei Municipal Tu Cheng Hospital, Chang Gung Memorial Hospital, New Taipei City 23656, Taiwan; mp0667@cgmh.org.tw; 2 Division of Gastroenterology, Department of Pediatrics, Chang Gung Memorial Hospital, Taoyuan City 33305, Taiwan; 3 Department of Pediatrics and Liver Research Center, Chang Gung Memorial Hospital, Taoyuan City 33305, Taiwan

**Keywords:** *Helicobacter pylori*, child, treatment, antibiotic resistance

## Abstract

*Helicobacter pylori* infection can cause gastritis, gastric or duodenal ulcers, mucosa-associated lymphoid tissue lymphoma, gastric cancer, and extra-gastrointestinal manifestations. Ideal treatment should be guided by antibiotic susceptibility testing. However, this is not feasible in many regions, so the treatment generally relies on clinical experience and regional culture sensitivity profiles. We aimed to integrate the treatment of pediatric *H. pylori* infection through a systematic literature review. Databases including PubMed, Cochrane Library, EMBASE, and Scholar were searched using terms containing (*Helicobacter* OR *Helicobacter pylori* OR *H. pylori*) AND (child OR pediatric) for all relevant manuscripts and guidelines, published from January 2011 to December 2021. The eradication rate for pediatric *H. pylori* infection was not satisfactory using triple therapy, sequential therapy, concomitant therapy, bismuth-based quadruple therapy, or adjuvant therapy with probiotics as the first-line therapy. Most therapies could not achieve the recommended eradication rate of >90%, which may be attributed to varying regional antibiotic resistance and possible poor children’s compliance. More studies are required to establish a best practice for pediatric *H. pylori* infection treatment.

## 1. Introduction

*Helicobacter pylori* (*H. pylori*) is a gram-negative bacterium, first isolated by Warren and Marshall in 1983 on the surface of the stomach [1,2]. *H. pylori* colonize gastric mucosa and could produce urease-dependent ammonia locally, raising the stomach pH, which promotes bacterial survival and solubilizes the mucous gel to facilitate bacterial motility [3]. *H. pylori* infection can cause gastritis, gastric or duodenal ulcers, mucosa-associated lymphoid tissue (MALT) lymphoma, and gastric cancer especially adenocarcinoma [1,4]. In addition, extra-gastrointestinal manifestations were reported, including iron deficiency anemia [5], chronic immune thrombocytopenic purpura, and impaired growth [6]. An inverse association was found between *H. pylori* infection and inflammatory bowel disease [7].

The global prevalence of *H. pylori* infection in adults was significantly higher than in children (48.6% vs. 32.6%, respectively), after an analysis of 410,879 participants from 73 countries on 6 continents [4]. A similar condition was also reported in China (adults 46.1% vs. children and adolescents 28.0%) [8]. The prevalence of pediatric *H. pylori* infection in Taiwan has varied in the past decades and in different areas. The declining prevalence rate between 1993 and 2012 was shown in a study conducted by Yeung et al., explained by urbanization and public health education regarding proper sanitation and food handling [9].

“Test and treat” strategy for *H. pylori* infection in children is not recommended [5,10] since no substantial evidence has been documented regarding the health benefits of treatment to eliminate *H. pylori* infection in children [11]. Testing for *H. pylori* is suggested in children with gastric or duodenal ulcers and when investigating causes of chronic immune thrombocytopenic purpura. Still, it is not recommended in children with functional abdominal pain, short stature, or as part of the initial investigation in children with iron deficiency anemia before other causes have been excluded [10]. It is still controversial in treating children whose first-degree relatives have gastric cancer or who have biopsy-proven *H. pylori* infection with non-ulcer dyspepsia [12]. The possibility of spontaneous eradication of *H. pylori* in infants and young children has been pointed out in some studies [13].

The preferable diagnosis was performed by endoscopy with biopsy-based methods. The cure of *H. pylori* after treatment can be assessed using the ^13^C-urea breath test (UBT) or stool antigen test at least 4 weeks after therapy [10,14].

The Joint ESPGHAN and NASPGHAN 2016 guidelines recommend a triple combination of PPI-CA (proton pump inhibitor, clarithromycin, and amoxicillin) for 14 days as the preferred regimen for *H. pylori* infection in children if the strain is known to be susceptible to clarithromycin [10,14]. However, if culture and antibiotic susceptibility testing for *H. pylori* are not available, the treatment may rely on clinical experience and regional antimicrobial susceptibility profiles. When the first-line treatment fails, culture with antibiotic sensitivity testing or polymerase chain reaction (PCR) or fluorescence in situ hybridization (FISH) on previously obtained paraffin-embedded biopsies should be performed to guide the subsequent therapy [15]. Yet, low adherence to the 2016 updated ESPGHAN and NASPGHAN guidelines was revealed in a study by Bonilla et al. [16], but several limitations of the study were pointed out [14].

In addition, the available literature was sparse about the treatment choice for those who were non-responders to the first- or second-line regimen. Levofloxacin-based triple therapy, including levofloxacin, lansoprazole, and metronidazole, seemed safe and effective as third-line rescue therapy if lansoprazole, clarithromycin, and amoxicillin were used as the first-line therapy and bismuth subcitrate, lansoprazole, metronidazole, and doxycycline as second-line therapy in a prospective study [17]. However, some antibiotics are not licensed for children or restricted by the regional policy; for example, levofloxacin was not reimbursed by the National Institutes of Health in Taiwan to treat children. Therefore, treating refractory pediatric *H. pylori* infection is an ordeal due to limited therapeutic options. The antibiotic resistance rates also vary with regions, so whether the first-line therapy in the Western world is suitable for Asian children is uncertain.

Therefore, we aimed to integrate the treatment of pediatric *H. pylori* infection through a systematic literature review.

## 2. Methods

**Figure 1:**
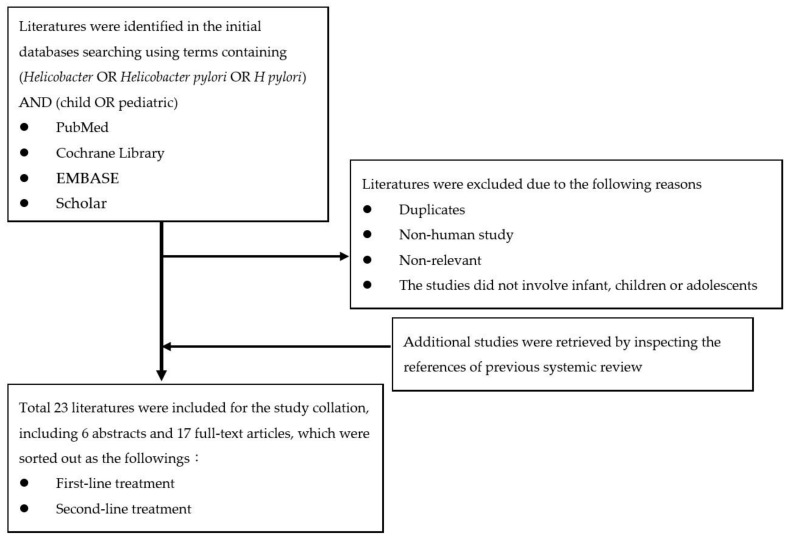
The flow chart of literature selection.

Databases including PubMed, Cochrane Library, EMBASE, and Scholar were searched using terms containing (*Helicobacter* OR *Helicobacter pylori* OR *H. pylori*) AND (child OR pediatric) for all relevant abstracts, manuscripts, and guidelines, published from January 2011 to December 2021. The non-human studies and manuscripts that did not involve children or adolescents were excluded. The association and management eligibility were initially screened based on the title and the abstract. Then a full-text assessment was performed with the evaluation of the references in the included studies to identify additional information. The flow chart of literature selection was shown in Figure 1.

The standard triple therapy mentioned in this review included a proton pump inhibitor, amoxicillin, and clarithromycin. The standard sequential therapy included a proton pump inhibitor with amoxicillin for 5 days followed by a proton pump inhibitor with clarithromycin and metronidazole for 5 days. The standard concomitant therapy included proton pump inhibitor, amoxicillin, metronidazole, and clarithromycin for 14 days.

If intention-to-treat analysis and per-protocol analysis were both stated in the relevant articles, the results of the per-protocol analysis were listed in the report to represent the eradication rate.

## 3. Results

### 3.1. First-Line Treatment

Current guidelines still recommend standard triple therapy as the first-line treatment for the eradication of *H. pylori* infection in children worldwide (Table 1). However, the eradication rate did not achieve the desired level in children.

**Table 1:** Recommended options for first-line therapy of *Helicobacter pylori* infection.

Published Year	Region	*Helicobacter pylori* Antimicrobial Susceptibility				
		Susceptible to CLA and MET	Resistant to MET, Susceptible to CLA	Resistant to CLA, Susceptible to MET	Resistant to CLA and MET	Unknown
2016 [10]	Europe	PPI-AMO-CLA 14 d		PPI-AMO-MET 14 d	PPI-high dose AMO-MET 14 d	
		Sequential therapy 10 d #	BIS-PPI-AMO-MET * 14 d #	BIS-PPI-AMO-MET * 14 d #	BIS-PPI-AMO-MET 14 d #	
					Concomitant therapy for 14 d *#	
Doses (morning dose/evening dose) of PPI and antibiotics are calculated based on the body weight and age:						
Body weight		15–24 kg		25–34 kg	>35 kg	
**PPI**		20 mg/20 mg		30 mg/30 mg	40 mg/40 mg	
		The PPI dose refers to esomeprazole and omeprazole and should be adapted if other PPIs are used.				
**AMO**		500 mg/500 mg		750 mg/750 mg	1000 mg/1000 mg	
**High dose AMO**		750 mg/750 mg		1000 mg/1000 mg	1500 mg/1500 mg	
**CLA**		250 mg/250 mg		500 mg/250 mg	500 mg/500 mg	
**MET**		250 mg/250 mg		500 mg/250 mg or 375 mg/375 mg	500 mg/500 mg	
Age		<10 years			>10 years	
**BIS**		262 mg QID			524 mg QID	
		Bismuth in the United States and Canada comes as bismuth subsalicylate.				
2019 [18]	Korea	PPI-AMO-CLA 14 d		PPI-AMO-MET 14 d	PPI-high dose AMO-MET 14 d	
		Sequential therapy 10 d #	BIS-PPI-AMO (TET)-MET 14 d *#	BIS-PPI-AMO (TET)-MET 14 d *#	BIS-PPI-AMO (TET)-MET 14 d *#	
					Concomitant therapy 14 d *#	
The doses of PPI, Amoxicillin, Clarithromycin, Metronidazole, and Bismuth are the same as stated above except for esomeprazole or omeprazole (1.5–2.5 mg/kg/d) are also mentioned.						
**TET**		500 mg QID (>12 years old, >40 kg)				
2020 [5]	Japan	PPI-AMO-CLA 7–14 d		PPI -AMO-MET 7–14 d		PPI-AMO-CLA 7–14 d
		Twice daily			Maximum daily dose (mg/day)	
**PPI**						
Lansoprazole		1.5 mg/kg/day			60	
Omeprazole		1.0 mg/kg/day			40	
Rabeprazole		0.5 mg/kg/day			20	
Esomeprazole		≥4 years old Body weight < 30 kg		20 mg/day	40	
		Bodyweight ≥ 30 kg		40 mg/day		
**AMO**		50 mg/kg/day			1500	
**CLA**		15–20 mg/kg/day			800	
**MET**		10–20 mg/kg/day			500	

# Alternative therapy. * In the case with penicillin allergy: if the strain is susceptible to CLA and MET, use standard triple therapy with MET in place of AMO; if the strain is resistant to CLA, then use bismuth-based therapy with tetracycline instead of AMO if >8 years old. Abbreviation(s): Proton pump inhibitor (PPI); Amoxicillin (AMO); Clarithromycin (CLA); Metronidazole (MET); Bismuth (BIS); tetracycline (TET).

As a first-line treatment for pediatric *H. pylori* infection, sequential therapy had a better eradication rate than triple therapy in recent studies. Huang et al. conducted a systematic review with meta-analysis to compare triple therapy with sequential therapy for treating *H. pylori* infection in children [19], which included all literature previously reviewed by Horvath et al. [20] except a retracted one. A Cochrane review including 44 randomized controlled trials discussing the issue with only six studies addressing children; however, one study [21] was excluded as the triple therapy using metronidazole instead of clarithromycin [22]. In addition, another study [23] included in the meta-analysis by Huang et el. compared concomitant therapy to sequential therapy instead of standard triple therapy. Additionally, some data reported by Baysoy et al. overlapped with those in the study by Huang et al. Table 2 lists the details of the literature included in this review except for one original article [24], which was not available; therefore, the data was extracted from a systematic review with a meta-analysis collated by Gatta et al. [25]. The treatment dosage mentioned in the associated articles is listed in Table 3. Due to the heterogenicity of the study design, treatment regimen, and follow-up methods, a meta-analysis of these studies was not performed.

**Table 2:** Eradication rates of first-line treatment for pediatric *H. pylori* infection.

Study	Region	Study Period	Follow-Up Case Number	Treatment	Eradication Rate
Francavilla et al., 2005 [21]	Italy	2002 to 2004	74 (Age 3.3–18 years; median age 12.3 years)	Triple therapy for 7 days OME + AMO + MET	75.7% (28/37)
				Sequential therapy for 10 days OME + AMO for 5 days followed by OME + CLA + TIN for 5 days	97.3% (36/37)
The method that detects the eradication of *H. pylori*: ^13^C-urea breath test, at least 4 weeks after the end of therapy					
Lerro et al., 2006 [26] (abstract)	Italy	Not available	25 (Median age 12.3 years)	Triple therapy for 7 days OME + AMO + TIN	80% (20/25)
			25 (Median age 11.9 years)	Sequential therapy for 10 days OME + AMO for 5 days followed by OME + CLA + TIN for 5 days	92% (23/25)
The method that detects the eradication of *H. pylori*: ^13^C-urea breath test, 6 weeks after the end of therapy					
Hurduc et al., 2007	Romania	Not available	135	Triple therapy for 7–14 days * PPI + 2 antibiotics (type of medication was not reported)	80% (36/45)
				Sequential therapy for 10 days * OME + AMO for 5 days followed by OME + CLA + TIN for 5 days	86.7% (39/45)
Lu et al., 2010 [27]	China	2006 to 2009	33 (Mean age 10.2 ± 2.8 years)	Standard triple therapy for 10 days OME + AM + CLA	78.8% (26/33)
			38 (Mean age 10.7 ± 2.4 years)	Sequential therapy for 10 days OME + AMO for 5 days followed by OME + CLA+ TIN for 5 days	94.7% (36/38)
The method that detects the eradication of *H. pylori*: ^13^C-urea breath test, at least 4 weeks after the end of therapy					
Anania et al., 2011 [23] (abstract)	Italy	Not available	15 (Age 5.8–16.7 years; median age 11 years)	Concomitant therapy for 5 days * OME + AMO + CLA + TIN	93.3% (14/15)
			15 (Age 4.8–14.1 years; median age 7.6 years)	Sequential therapy for 10 days * OME + AMO for 5 days followed by OME + CLA + TIN for 5 days	86.7% (13/15)
The method that detects the eradication of *H. pylori*: ^13^C-urea breath test, 8 weeks after the end of therapy					
Bontems et al., 2011 [28]	Belgian, France, Italy	2007 to 2009	150 (Median age 10.4 years)	Standard triple therapy for 7 days OME + AMO + CLA or MET for CLA-resistant strains	80.8% (59/73)
				Standard sequential therapy for 10 days OME + AMO for 5 days followed by OME + CLA + MET for 5 days	88.3% (68/77)
The method that detects the eradication of *H. pylori*: ^13^C-urea breath test, at least 8 weeks after the end of therapy					
Albrecht et al., 2011 [29]	Poland	2006 to 2009	103 (Age 3 to 18 years)	Standard triple therapy for 7 days + Placebo for 3 days OME + AMO + CLA for 7 days followed by placebo for 3 days	68.6% (35/51)
				Sequential therapy for 10 days OME + AMO for 5 days followed by OME + CLA + TIN for 5 days	86.5% (45/52)
The method that detects the eradication of *H. pylori*: ^13^C-urea breath test, 6–8 weeks after the end of therapy					
Liu et al., 2011 [30] (abstract)	China	Not available	100	Standard triple therapy for 10 days * OME + AMO + CLA	69.0% (33)
				Triple therapy for 10 days * OME + AMO + MET	76.7% (33)
				Standard sequential therapy for 10 days * OME + AMO for 5 days followed by OME + CLA + MET for 5 days	91.2% (34)
The method that detects the eradication of *H. pylori*: ^13^C-urea breath test, 4 weeks after the end of therapy					
Hong et al., 2012 [31]	Seoul, Korea	2004 to 2012	62 (Age 3.1–16.6 years; mean age 11.0±3.2 years)	Standard triple therapy for 14 days OME + AMO + CLA	67.7% (42/62)
			56 (Age 2.7–18.6; mean age 11.0±3.3 years)	Bismuth-based quadruple therapy for 7 days OME + AMO + MET + BIS citrate	83.9% (47/56)
The method that detects the eradication of *H. pylori*: ^13^C-urea breath test, 4 weeks after the end of therapy					
Huang et al., 2012 [32] (abstract)	Not available	Not available	199	Triple therapy for 7 days OME + AMC + CLA	71.4%
				Triple therapy for 10 days	67.3%
				Triple therapy for 14 days	82.0%
				Sequential therapy for 10 days OME + AMC for 5 days followed by OME + CLA + MET for 5 days	90.2%
The method that detects the eradication of *H. pylori*: ^13^C-urea breath test, 4 weeks after the end of therapy					
Hojsak, et al., 2012 [33]	Croatia	2001 to 2010	186 (Age 1.08–18.8 years; median age 12.9 years)	Triple therapy for 7–10 days PPI 1–2 mg/kg/day + AMO + MET or CLA	81.2% (151/186)
The method that detects the eradication of *H. pylori*: urea breath test or repeated endoscopy with culture					
Huang et al., 2013 [34]	China	2008 to 2010	318 (Age 3–16 years)	Standard triple therapy for 7 days OME + AMO + CLA	70.9% (73/103)
				Standard triple therapy for 10 days	77.8% (84/108)
				Standard sequential therapy for 10 days OME + AMO for 5 days followed by OME + CLA + MET for 5 days	89.7% (96/107)
The method that detects the eradication of *H. pylori*: stool antigen test, 4 weeks after the end of therapy					
Ali Habib HS et al., 2013 [35]	Jeddah, Saudi Arabia	Not available	16 (Age 12–15 years, male)	Standard triple therapy for 10 days rabeprazole + AMO + CLA	55.6% (5/9)
				Sequential therapy for 10 days rabeprazole + AMO for 5 days followed by rabeprazole + CLA + TIN for 5 days	57.1% (4/7)
The method that detects the eradication of *H. pylori*: ^14^C-urea breath test, 6 weeks after the end of therapy					
Laving et al., 2013 [36]	Kenya	2007	71 (Age 2–16 years; mean age 8.9 years)	Standard triple therapy for 10 days OME + AMO + CLA	48.9% (22/45)
				Sequential therapy for 10 days OME + AMO for 5 days followed by OME + CLA + TIN for 5 days	84.6% (22/26)
The method that detects the eradication of *H. pylori*: a stool antigen test and/or a repeat histology obtained at repeat endoscopy, 6 weeks after the end of therapy					
Baysoy et al., 2013 [37]	Turkey	2008 to 2010	61 (Age 4–18 years)	Standard triple therapy for 14 days LAN + AMO + CLA	54.2% (13/24)
				Sequential therapy for 10 days LAN + AMO for 5 days followed by LAN + CLA+ ORN for 5 days	48.6% (18/37)
The method that detects the eradication of *H. pylori*: ^13^C-urea breath test, 6–8 weeks after the end of therapy					
Kutluk et al., 2014 [38]	Turkey	2011	136 (Age 3–18 years)	Standard triple therapy for 10 days LAN + AMO + CLA	55.7% (39/70)
				Standard sequential therapy for 10 days LAN + AMO for 5 days followed by LAN + CLA and MET for 5 days	56.1% (37/66)
The method that detects the eradication of *H. pylori*: ^13^C-urea breath test, 4–6 weeks after the end of therapy					
Schwarzer, et al., 2016 [39]	European, a registry from nine European centers	2009 to 2011	209 (Age 3.1– 17.9 years)	Standard sequential therapy for 10 days ESO + AMO for 5 days followed by ESO + CLA + MET for 5 days (Dosage was chosen depending on weight: 15–24 kg, 25–34 kg, >35 kg)	80.4% (168/209)
The method that detects the eradication of *H. pylori*: ^13^C-urea breath test, by upper endoscopy with culture and histology, and/or by a monoclonal stool antigen test, 8–12 weeks after the end of therapy					
Zhou et al., 2020 [40]	China	2017 to 2018	228 (Age 6– 18 years)	Standard triple therapy for 14 days OME + AMO + CLA	74.1% (43/58)
				Sequential therapy for 14 days OME + AMO for 7 days followed by OME + CLA + MET for 7 days	69.5% (41/59)
				Bismuth-based quadruple therapy for 14 days OME + AMO + MET + elemental BIS	89.8% (53/59)
				Standard concomitant therapy for 14 days OME + AMO + CLA + MET	84.6% (44/52)
The method that detects the eradication of *H. pylori*: ^13^C-urea breath test, at least 4 weeks after the end of therapy					

* Dosage not reported; Abbreviation(s): Proton pump inhibitor (PPI); Omeprazole (OME); Lansoprazole (LAN); Esomeprazole (ESO); Amoxicillin (AMO); Amoxicillin-clavulanate (AMC); Clarithromycin (CLA); Metronidazole (MET); Tinidazole (TIN); Ornidazole (ORN); Bismuth (BIS); all drugs were given twice daily except the usage in Zhou et al., 2020: OME was given once or twice a day, AMO was given three times or four times a day, MET was given twice or three times a day, and BIS was given twice or three times a day.

#### 3.1.1. Triple Therapy

Overall, the eradication rates with standard triple therapy for 7 days were 68.6–80.8% [28,29,34], for 10 days, 48.9–78.8% [27,30,34,35,36,38], for 14 days, 54.2–74.1% [31,37,40]. The eradication rates with a different regimen using metronidazole instead of clarithromycin for 7 days and 10 days were 75.7% [21] and 76.7% [30], respectively. The eradication rate of one study using tinidazole instead of clarithromycin for 7 days was 80% [26].

#### 3.1.2. Sequential Therapy

The eradication rates with standard sequential therapy for 10 days were 80.4–91.2% [28,30,34,39], except for one study with a low eradication rate of 56.1% [38], in which the clarithromycin resistance rate was 25.7%. The eradication rate with standard sequential therapy for 14 days in one prospective study was only 69.5%, lower than standard triple therapy with an eradication rate of 74.1% [40]. The eradication rate with a different regimen using tinidazole instead of metronidazole were 84.6–97.3% [21,23,24,26,27,29,36], except for one study with a low eradication rate of 57.1% [35]. However, the participants in that study were asymptomatic and diagnosed with positive *H. pylori* immunoglobulin G and urea breath test, which was contrary to the recommendation against “test and treat”. One study using amoxicillin-clavulanate instead of amoxicillin in triple therapy and sequential therapy had similar results as the standard therapy mentioned above [32]. Only one study stated a higher eradication rate by standard triple therapy than sequential therapy using ornidazole instead of metronidazole, albeit with low rates in both (54.2% vs. 48.6%, *p* > 0.05) [37].

Bontems et al., tailored the therapy according to the antimicrobial susceptibility testing. Subgroup analysis indicated that eradication rates tended to be higher using the sequential treatment except for children harboring CLA-resistant strains with a per-protocol eradication rate of 80% in triple therapy and 64% in sequential therapy [28].

#### 3.1.3. Concomitant Therapy

The eradication rate of concomitant therapy regimen using omeprazole, amoxicillin, clarithromycin, and metronidazole for 14 days was 84.6% in one recent study [40]. The eradication rate of concomitant therapy for 5 days with a different regimen using tinidazole instead of metronidazole was 93.3%, higher than 86.7% in sequential therapy for 10 days also using tinidazole in one study [23].

#### 3.1.4. Bismuth-Based Quadruple Therapy

Bismuth-based quadruple therapy, including a proton pump inhibitor, amoxicillin, metronidazole, and bismuth citrate, has been reported in one study with an eradication rate of 83.9%, significantly higher than 67.7% in standard triple therapy [31]. The eradication rate of bismuth-based quadruple therapy could reach 89.8%, which is superior to 74.1% of standard triple therapy in another study [40].

#### 3.1.5. Adjuvant Therapy with Probiotics

Some studies have proposed that probiotics can inhibit *H. pylori* by immunological and non-immunological mechanisms, including regulating pro-inflammatory cytokines in the gastric mucosa, increasing local IgA concentration, and secreting antibacterial substances such as lactic acid, short-chain fatty acids, hydrogen peroxide and bacteriocin [41]. A tendency of decreasing specific anti-*H. pylori* IgG antibodies was found with probiotics use in an animal model, yet not to a statistically significant level [42].

A review included eight studies comparing the group treated with antibiotics to those using with same antibiotics therapy plus probiotics. Most of them imply a benefit of the probiotics, but only two studies reached a significant difference [43]. One randomized, double-blind study addressed that the probiotics group experienced less frequent diarrhea, nausea, or vomiting during eradication therapy and a better eradication rate (30/33, 90.9%) compared to the placebo group (23/33, 69.7%) [44]. A meta-analysis including five studies with 484 pediatric patients indicated that Lactobacillus-supplemented triple therapy could increase the eradication rate by approximately 13% (84.0% vs. 71.4%) and reduce the incidence of therapy-related diarrhea [45].

Standard sequential therapy includes a proton pump inhibitor with amoxicillin for 5 days followed by a proton pump inhibitor with clarithromycin and metronidazole for 5 days.

### 3.2. Second-Line Treatment

If eradication fails after first-line therapies, an endoscopy should be performed to obtain specimens for *H. pylori* culture and antibiotic susceptibility tests to tailor the subsequent therapy [5,10,18,46]. The current recommendation of second-line therapy for pediatric *H. pylori* infection in Korea is the same as that in the Joint ESPGHAN and NASPGHAN 2016 guidelines (Table 4). Whereas the JSPGHAN 2020 guidelines recommend that a proton pump inhibitor-based triple regimen with amoxicillin and metronidazole for 7 days as second-line therapies if *H. pylori* strains are resistant to clarithromycin and first-line therapy fails [5].

There was scarce data concerning the efficacy of second-line therapy for the treatment of pediatric *H. pylori* infection. Only two conference abstracts [47,48] were found (Table 5). The former was a mono-center, non-randomized, retrospective study that enrolled naïve children with *H. pylori* infection treated with a sequential regimen, and the initial eradication rate was 82.6% (181/219). Among the 38 children who remained infected, 30 children received second-line treatment, with 24 of them using a tailored triple therapy according to antimicrobial susceptibility test and 6 of them using a repeated sequential therapy; however, eradication rates of both therapies were low (65% and 40%). There were no sufficient details described in the latter. However, the overall eradication rate was only 67.3% after second-line treatment with sequential treatment or triple therapy.

### 3.3. Adverse Event

Adverse events were mentioned in some studies, and most were resolved once the treatment was stopped. Bontems et al. reported abdominal pain found in 20% (sequential 24% vs. triple therapy 17%), diarrhea in 14% (12% vs. 16%), nausea in 6% (8% vs. 5%) and vomiting in 2% (4% vs. 0%) in pediatric patients while receiving *H. pylori* eradication therapy [28]. Another report on adverse events showed no significant differences between sequential and triple therapy [22]. The overall incidence of adverse events was 12.3%, including rash and diarrhea, with no significant difference in the incidence of adverse effects following standard triple therapy (12.1%), sequential therapy (6.8%), bismuth-based quadruple therapy (15.3%), and concomitant therapy (15.4%) in a Chinese study [40]. Black tongues and dark stool were observed in some children using bismuth-based therapy.

## 4. Discussion

Successful eradication of pediatric *H. pylori* infection depends on the knowledge of *H. pylori* susceptibility to antibiotics and adherence to treatment [39]. The desirable goal of first-line therapy is at least a 90% eradication rate. A high initial eradication rate will prevent the emergence of antibiotic-resistant strains [15,49]. However, unsatisfactory eradication rates were encountered in pediatric patients with *H. pylori* infection.

If the strain is known to be susceptible to clarithromycin, both the Joint ESPGHAN and NASPGHAN 2016 guidelines and the JSPGHAN 2020 guidelines recommend the triple combination PPI-CA (proton pump inhibitor, clarithromycin, and amoxicillin) for 14 days as the preferred regimen [5,10]. However, the eradication rates of standard triple therapy for 14 days were only 54.2–67.7%, which is inferior to 80.4 to 91.2% with standard sequential therapy for 10 days in most studies. In the 2016 Cochrane review, sequential therapy was more beneficial than standard triple therapy (76% vs. 64%) but still lower than the eradication rates in the adult population, sequential vs. triple (83% vs. 75%). Lower eradication rates in children than in adults using the same regimen have been reported before. Still, no well-founded explanation was addressed except for some assumptions such as different antibiotic susceptibility or adherence to therapy between children and adults [12]. The different eradication rates between sequential and triple therapy were more impressive in Europe than in Asia, Africa, and South America [22]. However, a relatively low eradication rate of sequential therapy below 70–80% was also reported in four articles [35,37,38,40], which can possibly be attributed to different regional *H. pylori* resistance rates as two of the articles were performed in Turkey. Another estimated reason is the risk of increasing resistance rate to sequential therapy in recent years as the study conducted by Zhou et al. was performed from 2017 to 2018. Whether prioritizing sequential therapy to triple therapy when antibiotic susceptibility testing is not available is worthy of consideration.

An alternative first-line therapy for pediatric *H. pylori* infection should be chosen according to the regional *H. pylori* resistance rate to clarithromycin and metronidazole. *H. pylori* resistance to antibiotics varies geographically and throughout the decades. A study in Zagreb showed *H. pylori* resistance to antibiotics in treatment-naïve pediatric patients: clarithromycin (11.9%), metronidazole (10.1%) and amoxicillin (0.6%) [33]. A study using a registry from nine European centers in 2015 reported antibiotic resistance rates: clarithromycin (17.7%) and metronidazole (18.6%) [39]. In Swedish children, 21% (46/222) of *H. pylori* strains were clarithromycin-resistant from 2005 to 2016 [50]. In a meta-analysis of six studies in Iranian children, the prevalence of resistance to clarithromycin, metronidazole, and ciprofloxacin was 12%, 71%, and 16%, respectively [51]. Much higher resistance to clarithromycin (50.9%), metronidazole (65.3%), but not amoxicillin (0.5%) was shown in children aged 3–15 years old in a Vietnamese study [52]. Overall, the resistance rate to clarithromycin ranged from 11.9% to 50.9%; metronidazole, from 10.1% to 71%; amoxicillin, from 0.5% to 0.6%; ciprofloxacin, 16%, from various regions and periods. Savoldi et al. conducted a systematic review with meta-analysis regarding the antibiotic resistance in *H. pylori* in World Health Organization (WHO) regions in 2018, including 178 studies and 65 countries, reported resistance rates to clarithromycin, metronidazole, and levofloxacin were 15% in all WHO regions [53]. In Taiwan, Lu et al. conducted a study to investigate the antimicrobial susceptibility of *H. pylori* isolated from children in southern Taiwan in the past two decades, and the overall antimicrobial resistance rates of clarithromycin and metronidazole were 22.9% and 21.4%, respectively; the dual resistance rate of clarithromycin and metronidazole was 10%, and the resistance rates of levofloxacin and amoxicillin were 8.3% and 2.9%, respectively [54]. Increasing resistance to clarithromycin, metronidazole, and dual resistance to clarithromycin/metronidazole were noted from the period 1998–2008 to the period 2009–2018, from 17.2% to 26.8%, 5.3% to 9.8%, and 6.9% to 12.2%, respectively [54]. Therefore, regional antibiotic resistance should be monitored longitudinally, especially in areas with declining or low eradication rates.

Ideal first-line treatment should be guided by antibiotic susceptibility testing. If *H. pylori* strains are clarithromycin-resistant, triple therapy with metronidazole and amoxicillin (PPI-MA) is recommended as first-line therapy in the Joint ESPGHAN and NASPGHAN 2016 guidelines [10] and the JSPGHAN 2020 guidelines [5]. However, if the antimicrobial susceptibility of the strain is unknown, the triple combination of PPI-CA for 14 days is still recommended in the JSPGHAN 2020 guidelines [5]. The Joint ESPGHAN and NASPGHAN 2016 guidelines recommend a 14-day bismuth-based therapy or high dose triple therapy with PPI-MA if bismuth is not available [10]. The Korean study by Hong et al. also found that bismuth-PPI-MA (metronidazole-amoxicillin) for 7 days was more effective than standard triple therapy [18,31]. Concomitant therapy is an alternative without susceptibility testing. Anania et al. applied concomitant therapy with PPI-amoxicillin-clarithromycin-tinidazole for 5 days and achieved a high eradication rate of 93.3%. Zhou et al. compared four different regimens and found only bismuth-based therapy and concomitant therapy showed higher eradication rates [40]. The association with eradication outcome and susceptibility to antimicrobial agents in treatment for children truly exists as the eradication rates were significantly lower with the standard sequential treatment in the case of clarithromycin-resistant strains compared with strains susceptible to both metronidazole and clarithromycin in the study (64% vs. 93%) conducted by Bontems et al. in 2011 [28].

The eradication rate for pediatric *H. pylori* infection is not satisfactory, whether using first-line or second-line therapies. Standard 10-day sequential therapy seemed more effective (88.3–91.2%) than standard 14-day triple therapy (54.2–74.1%), especially if the strains are susceptible to clarithromycin [28]. However, some studies showed contrary results [37,40]. Some studies showed the addition of probiotics in the treatment regimens mitigated the side effects and improved the eradication rate [43,44,45]. Still, the evidence is not strong enough to be routinely recommended in the society guidelines.

Intrafamilial spreading is an important route of *H. pylori* transmission [55]. Zhao et al. concluded that whole family-based *H. pylori* treatment could partially increase the eradication rate in children and reduce recurrence over a single-infected patient treatment strategy [56]. However, family-based eradication therapy needs more research to weigh the benefit and harm of antibiotic exposure to the host and the environment, including the influence on the gut microbiome.

## 5. Conclusions

The eradication rates for pediatric *H. pylori* infection, compared to adult populations, are not satisfactory, no matter using triple, sequential, concomitant, or bismuth-based quadruple therapies, and most studies could not achieve the goal of 90% or above. In addition, the efficacy data of second-line therapy is sparse. Otherwise, retreatment as double resistance strains using high dose amoxicillin plus metronidazole, bismuth-based therapy, or concomitant therapy for 14 days is recommended in current guidelines modified from adult guidelines. However, the treatment protocol still varies by area and is practiced by clinical experience without consensus. The regimen may show superior results compared to other regimens in some studies, but the contrary results may be disclosed in other studies. More studies are required to improve the eradication therapy for pediatric *H. pylori* infection. The best strategy is still tailored treatment guided by culture with antibiotic sensitivity test and regional data about the eradication rates of different therapies need to be created for the best policy.

## Figures and Tables

**Table 3:** Dosage of the treatment for pediatric *H. pylori* infection in different studies.

	Medication	Dosage	Study	Maximum Dose
PPI	OME	1 mg/kg/day	Francavilla et al., 2005 Lerro et al., 2006 Albrecht et al., 2011 Hong et al., 2012 Laving et al., 2013 Zhou et al., 2020	40 mg/day
		0.8 mg/kg/day	Lu et al., 2010	
		0.8–1.0 mg/kg/day	Huang et al., 2012 Huang et al., 2013	
		10 mg twice a day below 30 kg	Bontems et al., 2011	
		20 mg twice a day above 30 kg		
	LAN	1 mg/kg/day	Baysoy et al., 2013 Kutluk et al., 2014	30 mg/day
	Rabeprazole	40 mg/day	Ali Habib HS et al., 2013	
Antibiotics	AMO	50 mg/kg/day	Francavilla et al., 2005 Lerro et al., 2006 Bontems et al., 2011 Albrecht et al., 2011 Hojsak, et al., 2012 Hong et al., 2012 Baysoy et al., 2013 Laving et al., 2013 Kutluk et al., 2014 Zhou et al., 2020	2 g/day
		40 mg/kg/day	Lu et al., 2010	
		30 mg/kg/day	Huang et al., 2013	
		1 g/day	Ali Habib HS et al., 2013	
	AMC	50 mg/kg/day	Huang et al., 2012	
	CLA	15 mg/kg/day	Francavilla et al., 2005 Lerro et al., 2006 Lu et al., 2010 Bontems et al., 2011 Hong et al., 2012 Baysoy et al., 2013 Laving et al., 2013	1 g/day
		20 mg/kg/day	Albrecht et al., 2011 Hojsak, et al., 2012 Huang et al., 2012 Huang et al., 2013 Kutluk et al., 2014 Zhou et al., 2020	
		500 mg/day	Ali Habib HS et al., 2013	
	MET	15 mg/kg/day	Francavilla et al., 2005	
		20 mg/kg/day	Kutluk et al., 2014 Zhou et al., 2020	1 g/day
			Bontems et al., 2011 Hojsak, et al., 2012 Hong et al., 2012 Huang et al., 2012 Huang et al., 2013	1.5 g/day
	TIN	20 mg/kg/day	Francavilla et al., 2005 Lerro et al., 2006 Albrecht et al., 2011 Laving et al., 2013	1 g/day
		15 mg/kg/day	Lu et al., 2010	
		1 g/day	Ali Habib HS et al., 2013	
	ORN	30 mg/kg/day	Baysoy et al., 2013	
	BIS	bismuth citrate 8 mg/kg/day	Hong et al., 2012	
		elemental bismuth 6–8 mg/kg/day	Zhou et al., 2020	330 mg/day

Abbreviation(s): Proton pump inhibitor (PPI); Omeprazole (OME); Lansoprazole (LAN); Amoxicillin (AMO); Amoxicillin-clavulanate (AMC); Clarithromycin (CLA); Metronidazole (MET); Tinidazole (TIN); Ornidazole (ORN); Bismuth (BIS); all drugs were given twice daily.

**Table 4:** Recommended options for second-line therapy of *Helicobacter pylori* infection.

Published Year	Region	*Helicobacter pylori* Antimicrobial Susceptibility				
		Past Treatment Regimen	Susceptible to CLA and MET	Resistant to MET, Susceptible to CLA	Resistant to CLA, Susceptible to MET	Unknown
2016 [10]	Europe	PPI-AMO-CLA	PPI-AMO-MET	◎	-	◎
		PPI-AMO-MET	PPI-AMO-CLA	-	Treatment like double resistance (#)	
		Sequential therapy	◎	-	-	
Doses (morning dose/evening dose) of PPI and antibiotics are calculated based on the body weight and age:						
Body weight		15–24 kg		25–34 kg	>35 kg	
**PPI**		20 mg/20 mg		30 mg/30 mg	40 mg/40 mg	
		The PPI dose refers to esomeprazole and omeprazole and should be adapted if other PPIs are used.				
**AMO**		500 mg/500 mg		750 mg/750 mg	1000 mg/1000 mg	
**High dose AMO**		750 mg/750 mg		1000 mg/1000 mg	1500 mg/1500 mg	
**CLA**		250 mg/250 mg		500 mg/250 mg	500 mg/500 mg	
**MET**		250 mg/250 mg		500 mg/250 mg or 375 mg/375 mg	500 mg/500 mg	
Age		<10 years			>10 years	
**BIS**		262 mg QID			524 mg QID	
		Bismuth in the United States and Canada comes as bismuth subsalicylate.				
2019 [18]	Korea	PPI-AMO-CLA	PPI-AMO-MET	◎	-	◎
		PPI-AMO-MET	PPI-AMO-CLA	-	Treatment like double resistance (#)	
		Sequential therapy	◎	-	-	
The doses of PPI, Amoxicillin, Clarithromycin, Metronidazole, and Bismuth are the same as stated above except for esomeprazole or omeprazole (1.5–2.5 mg/kg/d) are also mentioned.						
**TET**		500 mg QID (>12 years old, >40 kg)				
2020 [5]	Japan	PPI-AMO-CLA	PPI-AMO-MET for 7 days			
		Twice daily			Maximum daily dose (mg/day)	
**PPI**						
Lansoprazole		1.5 mg/kg/day			60	
Omeprazole		1.0 mg/kg/day			40	
Rabeprazole		0.5 mg/kg/day			20	
Esomeprazole		≥4 years old Body weight < 30 kg		20 mg/day	40	
		Bodyweight ≥ 30 kg		40 mg/day		
**AMO**		50 mg/kg/day			1500	
**CLA**		15–20 mg/kg/day			800	
**MET**		10–20 mg/kg/day			500	

◎ Considering performing a second endoscopy and using a tailored treatment for 14 d or treatment like double resistance: PPI-high dose AMO-MET 14 d or BIS-based therapy or concomitant therapy for 14 d * (Table 1); in adolescents, levofloxacin or tetracycline may be considered (#); * For a recommended duration of 14 days; in the case of penicillin allergy: if the strain is susceptible to CLA and MET, use standard dose triple therapy with MET in place of AMO; if the strain is resistant to CLA, then use bismuth-based therapy with tetracycline instead of AMO if older than 8 years. Abbreviation(s): Proton pump inhibitor (PPI); Amoxicillin (AMO); Clarithromycin (CLA); Metronidazole (MET); Bismuth (BIS); tetracycline (TET).

**Table 5:** Eradication rates of second-line treatment for pediatric *H. pylori* infection.

Study	Region	Study Period	Follow-Up Case Number	Past Treatment Regimen	Treatment	Eradication Rate
Genis et al., 2013 [47] (abstract)	Belgium	2007 to 2011	25	Sequential regimen	Tailored triple therapy for 10–14 days	3/5 (60%)
					Repeated sequential regimen	13/20 (65%)
Kallirroi et al., 2019 [48] (abstract)	Belgium	2011 to 2018	52	Not available	Sequential treatment either a triple therapy (tailored when secondary antimicrobial susceptibility was available) with duration and dosage per local treatment protocols (which were changing over time)	35/52 (67.3%)

## Data Availability

The authors confirm that the data supporting the findings of this study are available within the article.
